# Sperm-disrupting activity of a root bark fraction of *Flueggea virosa* (Roxb. ex willd.) royle uncovered using LC-MS/MS profiling and functional evaluation

**DOI:** 10.3389/fphar.2026.1835308

**Published:** 2026-07-01

**Authors:** Kehongo M. Nyanguru, Caroline Maina, Mutiat Ibrahim, James Kuria, Ephantus Ndirangu, Sospeter Ngoci Njeru, Abdullahi Abdulraheem, Edwin Murungi, Moses Obimbo, Adeyemi O. Aremu, Aloys Mosima Osano, Samwel Cheruiyot, Margaret O. Ilomuanya, Peter Waweru Mwangi, Elizabeth Kigondu

**Affiliations:** 1 Department of Mathematics and Physical Sciences, Maasai Mara University, Narok, Kenya; 2 Center for Community Driven Research (CCDR), Kenya Medical Research Institute (KEMRI), Nairobi, Kenya; 3 Department of Chemistry, University of Cape Town, Cape Town, South Africa; 4 Department of Pharmacognosy, Faculty of Pharmacy, University of Lagos, Lagos, Nigeria; 5 Department of Medical Biochemistry, Kisii University, Kisii, Kenya; 6 Department of Human Anatomy and Physiology, University of Nairobi, Nairobi, Kenya; 7 Indigenous Knowledge Systems (IKS) Centre, Faculty of Natural and Agricultural Sciences, North-West University, Potchefstroom, South Africa; 8 School of Life Science, College of Agriculture, Engineering and Science, University of KwaZulu-Natal, Durban, South Africa; 9 Department of Pharmaceutics and Pharmaceutical Technology, Faculty of Pharmacy, University of Lagos, Lagos, Nigeria; 10 Department of Human Anatomy and Medical Physiology, University of Nairobi, Nairobi, Kenya

**Keywords:** Acrosin-related activity, Flueggea virosa, intravaginal contraception, LC-MS/MS profiling, rabbit model, sperm immobilization

## Abstract

**Introduction:**

While *Flueggea virosa* (Roxb. Ex Willd.) Royle root bark is traditionally used in several parts of East Africa for fertility regulation, its contraceptive potential has not been systematically investigated. Given the paucity of safe, reversible, non-hormonal contraceptives for women, this study evaluated a root bark fraction for sperm-disrupting, intravaginal contraceptive activity using functional assays and a rabbit proof-of-concept model.

**Methods:**

Following extraction and fractionation of authenticated root bark, the resulting extracts were evaluated for cytotoxicity in Vero cells and for disruption of human sperm functions by ascertaining immobilization, revival, viability, cervical mucus penetration and acrosin-related activity. Thereafter, chemical profiling of the most active fraction was performed using liquid chromatography-tandem mass spectrometry (LC-MS/MS) for descriptive annotation. Moreover, *in vivo* contraceptive efficacy and short-term local safety (10 days) were determined after intravaginal administration of the fraction in rabbits by histological examination of cervicovaginal tissues.

**Results:**

The methanolic fraction (KBLM) demonstrated the strongest activity, completely immobilizing sperm, abolishing sperm revival after washing, markedly reducing sperm viability, strongly inhibiting cervical mucus penetration and suppressing acrosin-related activity at 1.62 mg/mL. In a rabbit proof-of-concept experiment, a single intravaginal dose prevented pregnancy at all tested concentrations (3.9, 7.8, and 15.6 mg/mL). Besides, repeated intravaginal exposure for 10 days revealed absent-to-mild cervicovaginal changes. Chemical fingerprinting putatively annotated flavonoid-, tannin-, and phenolic-related features, including catechin, quercetin derivatives, rutin, corilagin, and kaempferol glycosides, as prominent features of KBLM.

**Conclusion:**

Our findings have demonstrated the sperm disruption activity of the methanolic fraction of *F. virosa* root bark. The observed disruption of multiple sperm functions, observed efficacy in a rabbit model and absent-to-mild short-term local tissue changes support additional preclinical investigations of the extract geared towards further functional characterization, extended safety testing, standardization, and potential formulation of an intravaginal contraceptive.

## 1 Introduction

Unintended pregnancies remain a persistent public health challenge. Globally, between 2015 and 2022, the annual number of unintended pregnancies was approximately 121 million, with roughly 257 million women who wished to avoid pregnancy lacking access to safe, modern contraceptive methods (Sedgh and Bearak, 2025). Adolescent girls in sub-Saharan Africa are acutely affected as revealed by a pooled analysis of performance monitoring and accountability surveys across eight African countries which reported a 33% prevalence of unintended pregnancy among girls aged 15–24 years, with rates ranging from 13% in Niger to 45.7% in Kenya (Onivogui et al., 2024). Current national data indicate that the rate of teenage pregnancy in some Kenyan counties is roughly 50% (Omwodo et al., 2025), underscoring both the unmet need for family-planning services and the uneven access to contraceptive options. Although hormonal contraceptives are broadly effective, many women decline them due to adverse effects such as weight gain, mood disturbances and cardiovascular risks (Mu and Kulkarni, 2022). With the copper intrauterine device (IUD) currently being the most accessible long-acting, reversible, non-hormonal option (Omwodo et al., 2025), development of novel non-hormonal agents targeting gamete maturation, sperm motility, capacitation, the acrosomal reaction and fertilization is a pressing priority (Howard and Benhabbour, 2023). Indeed, sperm-specific enzymes including hyaluronidases and Ca2+ ion channels have been highlighted as probable reversible molecular targets in a male-contraception study (Norcross et al., 2022).

Plant-derived natural products remain an important source of fractions and compounds with contraceptive activity. For instance, *Aegle marmelos* leaf extracts have been demonstrated to immobilise sperm and inhibit hyaluronidase activity (Gunasekaran et al., 2023) while Gendarusin A from *Justicia gendurussa* and flavonoids from *Terminalia chebula* have been reported to interfere with fertility-related endpoints (Ghosh et al., 2017). In rural East African communities, botanicals widely used to modulate fertility include *Flueggea virosa* (Roxb. Ex Willd.) Royle [Phyllanthaceae; white-berry bush], which grows along Kenyan riverbanks and savannahs and has multiple local names: lubwili (Bukusu), mukwamba (Giriama), kiptarpotich (Pokot), mkwamba (Swahili), and Mukururu (Mbeere) (Ojha et al., 2024). Despite its long-standing use, the anti-fertility effects of *F. virosa* have not been scientifically characterized. Phytochemical profiling of this species is useful for chemotaxonomic and standardization purposes (Souleymane et al., 2023). However, because several putative phenolic and flavonoid annotations may represent promiscuous assay-interfering (PAINS) compounds, the LC-MS/MS data are used here only for descriptive chemical fingerprinting (Bolz et al., 2021; Magalhães et al., 2021).

In this study, *in vitro* and *in vivo* assays were used to ascertain the effects of the methanolic fraction of *F. virosa* root bark on sperm functions by determining the immobilization, revival, viability, cervical mucus penetration and acrosin-related activity. Furthermore, LC-MS/MS profiling was performed solely for descriptive chemical characterization of the fraction. Altogether, our results experimentally demonstrate fraction-level sperm disrupting activity of *F. virosa* root bark and provide a starting point for further investigations to chemically standardize the fraction and validate whether any isolated constituents contribute to the observed fraction-level effects.

## 2 Methods and materials

### 2.1 Materials

All chemicals used in this study were purchased from Sigma-Aldrich/Merck (Darmstadt, Germany). Unless otherwise stated, reagents were of analytical grade. For extraction procedures, *n*-hexane, dichloromethane (DCM), ethyl acetate, and methanol of industrial grade were utilized; these solvents were distilled before use to ensure purity.

Vero cells (ATCC® CCL-81™) were obtained from the Center for Traditional Medicine and Drug Research (CTMDR), Kenya Medical Research Institute (KEMRI), originally sourced from the American Type Culture Collection (ATCC, Manassas, VA, United States). Cells were maintained in Dulbecco’s Modified Eagle Medium (DMEM, high glucose; Gibco/Thermo Fisher Scientific, Waltham, MA, United States) supplemented with 10% fetal bovine serum (FBS; Gibco/Thermo Fisher Scientific) and 1% penicillin-streptomycin.

Semen samples were collected from healthy adult donors at ABIMS Fertility and Andrology Clinic, Lagos, Nigeria, following ethical approval from the University of Lagos Research Ethics Committee (UNILAGREC/23/08/006) and the Kenya Medical Research Institute (KEMRI) Scientific and Ethics Review Unit (SERU) (KEMRI/SERU/CTMDR/2025/5099). Written informed consent was obtained from all participants prior to sample collection. Semen samples were processed within 1 h of collection and analyzed according to the World Health Organization (WHO) Laboratory Manual for the Examination and Processing of Human Semen, sixth Edition (2021), prior to experimental use (Björndahl and Kirkman Brown, 2022). Only samples that met the laboratory’s acceptance criteria for routine WHO-based semen assessment were utilised in the functional assays.

The root bark of *F. virosa* (Roxb. Ex Willd.) Royle [Phyllanthaceae] was collected from Kathuri Village (−0.764528° S, 37.683142° E), Mbeere South sub-county, Embu County, Kenya in July 2024. The plant was taxonomically identified and authenticated by Dr. Mathias at the National Museum of Kenya and a voucher specimen (KMKS003) deposited at the East African Herbarium (EAH), National Museum of Kenya, for future reference. The root bark samples were harvested sustainably from wild populations, placed in sterile polyethylene collection bags, and transported to KEMRI’s Centre for Traditional Medicine and Drug Research (KEMRI-CTMDR) for further processing. The collected root bark was manually sliced into small segments and spread in a single layer on raised racks at ambient laboratory conditions (23 °C ± 2 °C, 40%–60% relative humidity). Air-drying proceeded for approximately 4 weeks until the sample mass remained constant. The dried material was milled to a fine powder using an electric mill (Christy Model 8, Serial No. 51474). Powders were transferred into brown paper, labeled with collection details and voucher number, and stored in a moisture-free shelf awaiting extraction (Okpako et al., 2024; Nyanguru et al., 2025). The collection and use of *F. virosa* root bark complied with applicable institutional and national regulations governing research on medicinal plant materials in Kenya. The species is not listed under CITES. Plant collection was conducted with local authorization, and access and benefit-sharing obligations under the Nagoya Protocol were considered where applicable.

### 2.2 Extraction and preparation of plant extract/fraction

To obtain a broad-spectrum extract for preliminary screening (total methanolic extract), powdered root bark material (500 g) was macerated in methanol at room temperature for 72 h with intermittent shaking. Methanol was chosen because of its efficiency in extracting a wide range of secondary metabolites, including both polar and semi-polar compounds. After 72 h, the mixture was filtered through Whatman No. 41 filter paper, and the residue was re-extracted with a fresh portion of methanol for an additional 48 h. Filtrates were pooled and concentrated under reduced pressure at 50 °C using a rotary evaporator (BUCHI Labortechnik AG, Model R-300 EL, Serial No. 1100033194). The total methanolic extract (KBLMt) was transferred to pre-weighed vials, air-dried at room temperature for 14 days, and stored in an activated silica gel desiccator until a constant weight was achieved (Kigondu et al., 2016; Obakiro et al., 2022). To separate phytochemicals by polarity and facilitate bioassay-guided fractionation, a separate batch of powdered plant material (1.5 kg) was subjected to serial maceration with solvents of increasing polarity. Plant powder was first extracted with n-hexane (8,000 mL) for 3 days with periodic shaking, followed by filtration through Whatman No. 1 filter paper. The residue was re-extracted with the same solvent for two additional days, and the combined filtrates were concentrated under reduced pressure using a rotary evaporator (BUCHI Labortechnik AG, Model R-300 EL, Serial No. 1100033194). The n-hexane step targeted nonpolar compounds such as lipids, waxes, and terpenoids.

The dried plant residue was then sequentially re-extracted with dichloromethane (DCM), ethyl acetate (EtOAc), and finally methanol (MeOH), using the same procedure described for n-hexane. DCM was applied to recover additional non-polar to moderately non-polar metabolites, EtOAc was used to extract semi-polar constituents, and MeOH was employed to extract highly polar compounds. Each fraction was concentrated, dried at room temperature for 14 days, and further stored in a silica gel desiccator until constant weight (Kigondu et al., 2016; Njeru et al., 2015). The extraction yield was calculated as Equation 1:

$$\text{Percentage extracted}=\left(\frac{Massofextract}{Massofplantmaterial}\right)\times 100\%$$ (1)

### 2.3 Cytotoxicity

Vero cells were used for the preliminary general cytotoxicity screening for overt mammalian-cell toxicity and were not intended as a surrogate for vaginal epithelial safety. Samples were directly introduced into complete EMEM without an added organic vehicle; untreated medium served as the control condition. Cytotoxicity of the plant fractions were determined using the MTT assay, as described by Mosmann (1983) with minor modifications. Vero CCL-81 cells (African green monkey kidney cells; ATCC CCL-81) were cultured in Eagle’s Minimum Essential Medium (EMEM) supplemented with 10% fetal bovine serum (FBS; GIBCO, United States), 1.5% sodium hydrogen carbonate (LOBA Chemie, India), 1% GlutaMAX (GIBCO, United States), 1% penicillin–streptomycin (GIBCO, United States), and 1% HEPES buffer (Gold Biotechnology, United States). The cells were incubated at 37 °C in a humidified incubator with 5% CO_2_ and 98% relative humidity until 80% confluency was reached, after which they were subcultured for cytotoxicity testing (Mosmann, 1983).

At 80% confluency, cells were washed with phosphate-buffered saline (PBS; Sigma-Aldrich, United States) and detached using 0.25% trypsin-EDTA (Solarbio, China). Trypsinization was halted by adding complete EMEM, and cell viability was confirmed using trypan blue exclusion (Loba Chemie, India). The cells were counted with a hemocytometer and adjusted to a density of 1 × 10^5^ cells/mL. Aliquots of 1 × 10^4^ cells were seeded per well in 96-well plates (Biologix, China) and incubated overnight at 37 °C to allow cell attachment.

After 24 h, the medium was replaced with EMEM containing serial dilutions of the test samples (1.37–1000 μg/mL final concentrations). Following 48 h of exposure, 10 µL of MTT solution (5 mg/mL in PBS; Solarbio, China) was added to each well and incubated for 4 h. The supernatant was removed, and the formazan crystals were dissolved in 100 µL of DMSO (Finar Chemicals, India). Absorbance was read at 570 nm (Thermo Fisher Scientific, USA). All experiments were performed in triplicate (Mosmann, 1983; Van Meerloo et al., 2011). All assays were performed in triplicate, and results are reported as mean ± standard deviation (SD) from at least two independent biological replicates. Cell viability (%) was calculated as Equation 2;

$$\%\text{ Cell live }\left(\text{cell viability}\right)=\left(\frac{At-Ab}{Ac-Ab}\right)\times 100\%$$ (2)

where: At = test absorbance, Ab = blank, Ac = untreated control.

Half-maximal cytotoxic concentrations (CC_50_) were computed using nonlinear regression in GraphPad Prism 9.0. Statistical differences among fractions were analyzed by one-way ANOVA, with p < 0.05 considered significant (Kigondu et al., 2009; Kigondu et al., 2011).

### 2.4 Sperm immobilization assay

#### 2.4.1 Sperm immobilization

The sperm immobilization effect of the plant total methanolic extract and fractions was assessed using a modified version of the Sander and Cramer test (Berberian et al., 1965; Björndahl and Kirkman Brown, 2022). Liquefied human semen was adjusted to 60 × 10^6^ sperm/mL in physiological saline (0.9% NaCl). Stock extract solutions (20 mg/mL) of each plant sample were prepared in physiological saline and serially diluted to yield test concentration. For each test, 100 µL of extract solution at the desired concentration was rapidly mixed with 100 µL of the sperm suspension (extract: sperm, 1:1, v/v) for 10 s. Immediately thereafter, a 10 µL drop of the mixture was placed on a pre-warmed glass slide, covered with a coverslip, and examined under a microscope at ×20 magnification (Euromex Bioblue. Lab, S/N-EC 221750.) Spermatozoa that lost complete motility within 20 s of exposure to the extract were considered immobilized and selected for further motility revival testing. In each sample, motile and immotile sperm were counted in at least five random fields and expressed as a percentage of total spermatozoa. Negative controls consisted of sperm mixed with physiological saline alone, while positive controls were treated with nonoxynol-9 (N-9). All sperm-function assays were performed in triplicate for each test condition. Independent biological replicates represent separate semen samples collected from distinct healthy donors (n ≥ 2), with each donor providing a unique sample that was processed individually. Results are expressed as mean ± SD. No organic co-solvent was used in the sperm immobilization assay; accordingly, physiological saline served as both the negative control and the vehicle control.

#### 2.4.2 Sperm revival

The extract/fractions that demonstrated 100% inhibition of sperm motility were selected for the sperm motility revival evaluation to assess reversibility after treatment. Each concentration was washed twice with an extract-free Sil-Select Plus™ sperm washing medium to ensure that no phytochemicals remained and incubated at 37 °C for 30 min, examined, and subsequently re-incubated for an additional 60 min.

To observe sperm motility, 10 μL of the incubated mixture was placed on a microscopic slide, covered with a cover slip, and examined under a light microscope at ×40 magnification. The number of motile spermatozoa were counted after 30 min and again after 60 min of incubation. In each sample, motile sperm were counted in five random fields and expressed as a percentage of total spermatozoa. Reversibility of immobilization (revival) was calculated as the proportion of motile sperm post-wash relative to the initial control motility, as described by Sander-Cramer. All assays were performed in triplicate, and results are reported as mean ± standard deviation (SD) from at least two independent biological replicates (Berberian et al., 1965; Björndahl and Kirkman Brown, 2022).

### 2.5 Sperm function assay

#### 2.5.1 Sperm viability evaluation

Fractions that demonstrated complete sperm immobilization were further evaluated for sperm viability using eosin-nigrosin staining. Briefly, 100 μL of each plant extract at the indicated test concentration was mixed with 20 μL of liquefied semen for 10 s. Thereafter, 20 μL of treated sperm suspension was mixed with 20 μL of eosin-nigrosin stain, smeared onto clean glass slides, air-dried, and examined under light microscopy at ×40 magnification. For each slide, at least 200 spermatozoa were counted across five random fields. Unstained spermatozoa were scored as viable, whereas pink-stained spermatozoa were scored as non-viable. Physiological saline and nonoxynol-9 served as the negative and positive controls, respectively. Results are reported as mean ± SD from triplicate determinations obtained in at least two independent biological replicates (Kanna and Shetty, 2025). Unless otherwise stated, sperm-based functional assays used physiological saline as the vehicle, and saline alone served as the vehicle/negative control.

#### 2.5.2 Cervical mucus evaluation (capillary penetration test)

Simulated cervical mucus (SCM) was prepared using the Extended Hydration Process previously described by Burruano et al. (2002). Briefly, the powder was dissolved in 90% (v/v) of the total water volume in a glass beaker and mixed on a magnetic stirrer at 500 rpm for 5 min. The remaining SCM constituents were then incorporated in order of increasing mass and mixed at 500 rpm for 10–15 min at 25 °C. Finely powdered, deshelled Irvingia gabonensis seed was added slowly into the vortex while the mixture was heated from 40 °C to 80 °C for 15–20 min at 500 rpm, until a visible increase in viscosity indicated complete hydration. The dispersion was cooled to 25 °C, mucin was added and mixed to homogeneity, and the buffer salts (pre-dissolved in the remaining 10% of the total water) were incorporated. Cross-linking was initiated by adding sodium borate solution with continuous stirring. The pH was adjusted to 7.4 (Burruano et al., 2002). The final gel was allowed to equilibrate at room temperature and inspected to ensure an even, bubble-free texture prior to capillary loading.

The distance through which the most progressive spermatozoa migrated through SCM in the presence of the test plant extracts was evaluated. SCM was loaded into a microcapillary hematocrit tube by applying a vacuum to one end of the tube. The tube was then removed from the mucus, and the resulting strand of mucus was cut off. The tubes were sealed at both ends with parafilm and stored at −20 °C until use. Tubes containing approximately 40 mm of cervical mucus and free of bubbles or debris were thawed for use in the assay. Each tube was broken at the mucus interface and inserted for 30 min into a solution containing an effective concentration of plant extract, physiological saline (negative control), or N-9 (positive control). After 30 min, the mucus-containing tubes were removed from these solutions and reinserted into a second solution consisting of extract-semen or control-semen mixtures, followed by incubation at 37 °C for 60 min. After the incubation period, the capillary tubes were removed, and sperm migration into the mucus column was assessed microscopically by measuring the length of the tube traversed by the most advanced motile spermatozoon. Migration by spermatozoa in the extract–semen mixtures was reported as a percentage of the migration observed in the negative-control semen mixture (Equation 3) (Björndahl and Kirkman Brown, 2022; Katz et al., 1980).

$$\%\text{ Inhibition}=\left(\frac{L\_control-L\_treatment}{Lcontrol}\right)\times 100\%$$ (3)

where L_control and L_treatment represent mean penetration distances in negative control (normal saline) and extract-treated samples, respectively. All assays were performed in triplicate, and results are reported as mean ± standard deviation (SD) from at least two independent biological replicates.

#### 2.5.3 Acrosin inhibition activity

Acrosin-related proteolytic activity in treated sperm suspensions was estimated using a semen-based (2–10 × 106 spermatozoa per mL) colorimetric BAPNA assay, following Kennedy et al. (1989), with minor modifications. This assay was used as a functional screening method and was not designed to establish inhibition kinetics, target specificity, or direct binding to purified acrosin. Briefly, liquefied semen (2–10 × 10^6^ spermatozoa/mL) was layered over 11% Ficoll, centrifuged at 1,000 × g for 30 min, and the sperm pellet was resuspended in 100 μL of test extract, benzamidine-HCl (positive control), or physiological saline (negative control). The reaction was initiated by adding 1 mL of freshly prepared substrate-detergent mixture containing BAPNA and incubating for 3 h. Reactions were terminated with benzamidine-HCl, centrifuged, and absorbance of the supernatant was measured at 410 nm. Acrosin-related activity was expressed as Equation 4 μIU/10^6^ sperm and interpreted according to the criteria of Kennedy et al. (1989). All assays were performed in triplicate from at least two independent biological replicates.

$$\mu \text{IU}=\left(\frac{\left({OD}_{test}-{OD}_{control}\right)\times 1000000}{\frac{\left(9.9\times 180\right)}{totalvoumeofthesemen}\times NumberofspermlayerontoFicoll\left(permillion\right)}\right)$$ (4)

where ODtest is the optical density of the test samples or negative control, ODcontrol is the optical density of the positive control (benzamidine-HCl), and μIU is a micro-international unit (one IU of acrosin activity is defined as the amount of enzyme that hydrolyzes μmol of BAPNA/min at 23 °C) (Kennedy et al., 1989).

### 2.6 *In vivo* intravaginal evaluation

#### 2.6.1 Acclimatisation and dosage

Adult female New Zealand white rabbits (3–4 months old, weight 2–3 kg) were acclimatized for 14 days under standard laboratory conditions (12 h light/dark, *ad libitum* food/water). All procedures were approved by the KEMRI Animal Care and Use Committee and followed national guidelines. Dose selection was empirical and intended for proof-of-concept intravaginal testing. Because KBLM produced complete sperm immobilization at 1.62 mg/mL *in vitro*, the lowest rabbit dose was set above this threshold (3.9 mg/mL), while the middle and high doses (7.8 and 15.6 mg/mL) represented approximately 2-fold and 4-fold increases over the low dose to explore a preliminary local dose-response window. No pharmacokinetic, tissue-retention, or systemic exposure studies were performed; therefore, these doses should not be interpreted as PK-optimized or as defining a formal safety margin. Each dose group contained n = 5 rabbits; additional groups of n = 5 each received normal saline (negative control) or nonoxynol-9 (N-9) (positive control) (Castle et al., 1998; Fda and Cder, 2014). KBLM dosing solutions were prepared in physiological saline, and the saline-only group served as the vehicle/negative control. Animals were randomly allocated to the saline control, nonoxynol-9 control, and KBLM treatment groups (3.9, 7.8, and 15.6 mg/mL), with five female rabbits per group.

#### 2.6.2 *In vivo* efficacy evaluation

After 14 days of acclimatisation, each female rabbit received a single 2 mL intravaginal instillation of the assigned test solution via a soft catheter. After about 10 min, each doe was paired with a proven male for mating; successful copulation was confirmed by observing the male’s ejaculatory lock and dismount. Mated females were returned to individual cages. On gestational day 14 (peak implantation), animals were euthanized and the reproductive tract exposed. The primary endpoint was pregnancy status, assessed by the number of implantation sites or conceptuses in the reproductive tract. Conception rates (pregnant versus not pregnant) and mean implantation counts per group were recorded. This single-dose mating protocol is standard in rabbit contraceptive studies (Castle et al., 1998; Ensign et al., 2014).

#### 2.6.3 *In vivo* safety evaluation

A separate cohort of rabbits (n = 5 per group) received once-daily intravaginal dosing of the assigned test solution for 10 consecutive days, following FDA-recommended RVI (rabbit vaginal irritation) protocols. Animals were checked daily for clinical signs, body weight, feed intake, and local reactions (vaginal discharge, erythema, edema). After the final dose (day 10), animals were euthanized; a full gross necropsy was performed. The entire reproductive tract (cervicovaginal, uterus, and ovary) was collected and fixed in 10% neutral-buffered formalin. Any gross lesions were noted. A 10-day repeated-exposure period was chosen to provide an initial short-term local tolerability assessment within established rabbit vaginal irritation screening paradigms (Castle et al., 1998; D’Cruz and Uckun, 2003; Fda and Cder, 2014). Formalin-fixed reproductive tissues were processed, embedded, sectioned, and stained with hematoxylin and eosin. Slides were evaluated by a board-certified veterinary pathologist who was blinded to treatment allocation. Lesions were scored on a semiquantitative ordinal scale (0 = none, 1 = mild, 2 = moderate, 3 = marked) for key endpoints: epithelial integrity (degeneration/ulceration), submucosal inflammation (leukocyte infiltrate), edema, vascular congestion/hemorrhage, and necrosis. These criteria follow standard RVI scoring systems (Castle et al., 1998; D’Cruz and Uckun, 2003; Fda and Cder, 2014).

### 2.7 Descriptive chemical profiling of sperm-disrupting fractions using liquid chromatography–tandem mass spectrometry (LC-MS/MS)

The methanol (KBLM) and ethyl acetate (KBLE) fractions were subjected to LC-MS/MS analysis (Agilent MSD-XT) to decode their chemical profiles at the University of North Carolina, Greensboro, United States of America. This analysis was aimed at determining the probable chemical fingerprint of the fractions and not to assign bioactivity, pharmacological specificity or therapeutic relevance to individual metabolites. Briefly, the fractions were re-dissolved in methanol, sonicated, and diluted with the mobile phase to a concentration of 5 mg/mL (Hussain et al., 2018). Chromatographic separation was performed on an Agilent Infinity LC system equipped with an Eclipse C18 column (4.6 × 100 mm, 3.5 µm) maintained at 25 °C ± 1 °C. The mobile phase consisted of water (B) and acetonitrile (A) and was delivered in a gradient: 10%–90% B at 0 min, 30%–70% B from 0 to 2 min, 35%–65% B from 2 to 5 min, 40%–60% B from 5 to 7 min and 100% B from 7 to 10 min, followed by a 2-min re-equilibration. The flow rate was 0.16 mL/min and the injection volume 1 µL. In the instrument’s “Top-6 peak report” used for the extract, the mobile phase was described more broadly as an acetonitrile gradient increasing from 5% to 95% over 10 minutes with electrospray ionization detection. The above gradient, therefore, matches this range but provides specific steps. The LC column effluent was directed to an Agilent 6410 triple-quadrupole mass spectrometer fitted with an electrospray ionization source. Data were acquired in full-scan mode (100–1200 m/z) and by multiple-reaction monitoring (MRM) for marker compounds. The mass spectrometer operated in negative and positive-ion mode with the gas temperature at 300 °C, drying-gas flow 10 L/min, nebuliser pressure 30 psi, cone voltages of 110 V–160 V, and collision energies of 15 eV and 18 eV, respectively. Although under these conditions’ prominent chemical features of the *F. virosa* fractions were determined as illustrated by the consistent retention times and mass spectra, the annotations are putative and require confirmation using authentic standards (Hussain et al., 2018).

### 2.8 Statistical analysis

All data processing and statistical analyses were performed in GraphPad Prism 9.0 (GraphPad Software, San Diego, CA, United States). For the cytotoxicity assay, concentration-response curves were fitted by nonlinear regression for the estimation of the CC_50_ values. For the *in vitro* sperm-function assays, results are presented as mean ± SD from triplicate determinations obtained in at least two independent biological replicates, and comparisons among groups were analyzed by one-way ANOVA where appropriate, with p < 0.05 considered statistically significant. Because the rabbit efficacy and safety studies were conducted as small proof-of-concept experiments (n = 5/group), *in vivo* outcomes are presented primarily descriptively as pregnant/total, mean ± SD, and median [range] (Cordova et al., 2023).

### 2.9 Ethical considerations

This study was approved by the Kenya Medical Research Institute Scientific and Ethics Review Unit (KEMRI/SERU/CTMDR/2025/5099). Semen samples were provided by healthy adult volunteers who gave written informed consent after a full briefing on study aims and procedures. Donor anonymity was preserved through coded sample labels, and participants retained the right to withdraw at any point without consequence. All work with human material adhered to the Declaration of Helsinki and local regulations governing research on human specimens. Ethical approval number (UNILAGREC/23/08/006) from the University of Lagos.

## 3 Results

### 3.1 Percentage extraction and cytotoxicity

The root bark of *F. virosa* (KBL) produced fractions with yields of 2.3% in hexane (KBLH), 4.0% in dichloromethane (KBLD), 4.5% in ethyl acetate (KBLE), and 10.3% in methanol (KBLM). The total methanolic extract (KBLMt) accounted for 23.93% of the dry material (Table 1).

**TABLE 1:** Cytotoxic effects (CC_50_ values) and extraction percentages of F. virosa extracts and solvent fractions in Vero cells.

Extract/Fraction	% yield	CC_50_
Hexane fraction (KBLH)	2.30	-
Dichloromethane (KBLD)	4.00	2129.93 ± 35.6
Ethyl acetate fraction (KBLE)	4.50	644.42 ± 28.9
Methanol fraction (KBLM)	10.30	536.41 ± 24.7
Total methanolic extract (KBLMt)	23.93	-

Statistical analysis using one-way ANOVA, revealed significant differences among fractions (p < 0.05), indicating concentration-dependent cytotoxic responses. The hexane (KBLH) and total methanolic extract (KBLMt) fractions were not tested. CC_50_: concentration causing 50% loss of Vero-cell viability.

The cytotoxic effects of KBLM, KBLE, and KBLD were evaluated in Vero cells using the MTT assay; KBLH was excluded because of insolubility (Table 1). Extracts were tested over a concentration range of 4.115–3000 μg/mL, and their respective 50% cytotoxic concentrations (CC_50_) were determined. The results revealed differential cytotoxic profiles among the extracts (Figure 1). All tested fractions showed low cytotoxicity in Vero cells (CC_50_ > 500 μg/mL), providing preliminary evidence of limited general cytotoxicity under the conditions tested, based on National Cancer Institute criteria (Anywar et al., 2022; Indrayanto et al., 2021; Weerapreeyakul et al., 2012).

**FIGURE 1:**
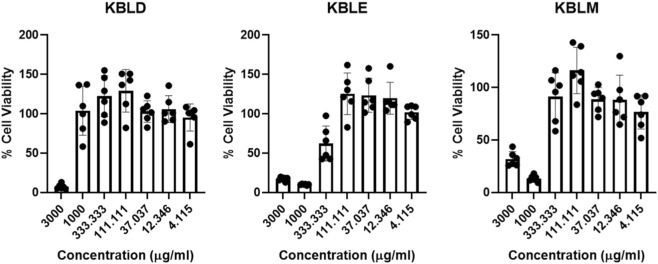
Cytotoxicity evaluation of F. virosa fractions in Vero cells. KBLM (methanolic fraction; CC_50_ = 536.41 ± 24.7 μg/mL), KBLD (dichloromethane fraction; CC_50_ = 2129.93 ± 35.6 μg/mL), and KBLE (ethyl acetate fraction; CC_50_ = 644.42 ± 28.9 μg/mL) were assessed across concentrations ranging from 4.115 to 3000 μg/mL*.*

### 3.2 Sperm immobilization, sperm revival, and viability assay

#### 3.2.1 Sperm immobilization

The total methanolic extract (KBLMt), ethyl acetate fraction (KBLE; semi-polar), and methanolic fraction (KBLM; polar) of *Flueggea virosa* root bark were evaluated for sperm-immobilizing activity across concentrations of 0.312–200 mg/mL (Figure 2). All samples produced concentration-dependent reductions in sperm motility. KBLMt showed a clear dose-response pattern, reaching >75% sperm immobilization with 25% non-progressive sperm at 2.5 mg/mL (100% total immobilization), which was defined as its minimum effective concentration (MEC). Complete spermicidal activity (100% loss of viability) was achieved at 100 mg/mL (Figure 2A). KBLE exhibited moderate activity, producing >73% sperm immobilization with 27% non-progressive sperm at 25 mg/mL (100% sperm immobilization), while complete spermicidal activity was only observed at 200 mg/mL (Figure 2B). KBLM was the most active fraction, inducing >77% sperm immobilization with 23% non-progressive sperm at 1.62 mg/mL (100% sperm immobilization). Notably, complete spermicidal activity (100%) occurred at 12.5 mg/mL (Figure 2C). Representative micrographs support these endpoints (Figure 2): panels E–G show fields at each MEC with no motile spermatozoa, whereas the negative control (0.9% NaCl; panel D) shows actively motile sperm. Positive control (nonoxynol-9) exhibited complete sperm immobilization at 1 mg/mL.

**FIGURE 2:**
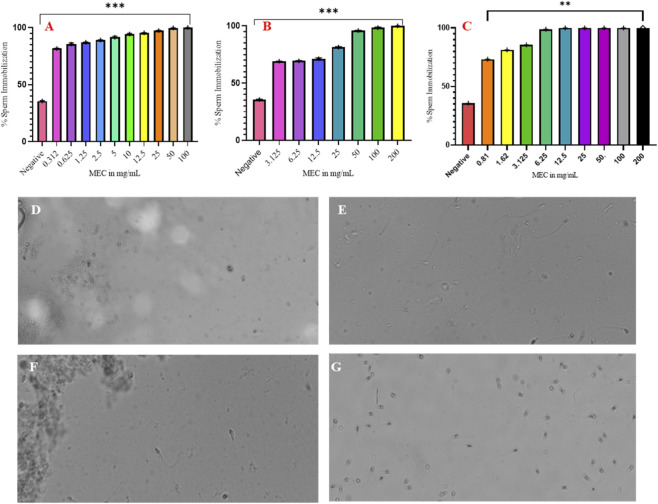
Sperm-immobilizing activity of Flueggea virosa root bark extracts. **(A)** Total methanolic extract (KBLMt), **(B)** ethyl acetate fraction (KBLE), and **(C)** methanolic fraction (KBLM) were evaluated at 0.312–200 mg/mL. KBLMt achieved >75% immobilization at 2.5 mg/mL and 100% at 100 mg/mL; KBLE produced >73% immobilization at 25 mg/mL and 100% at 200 mg/mL; KBLM induced >77% immobilization at 1.62 mg/mL and 100% at 12.5 mg/mL. **(D)** 0.9% NaCl control showing actively motile spermatozoa. **(E–G)** Representative micrographs at respective MECs showing absence of motile sperm.

#### 3.2.2 Sperm revival and viability

The KBLMt and KBLM of *F. virosa* were tested at concentrations between 1.62 and 100 mg/mL for sperm revival and viability (Table 2). There was no sperm revival after 1 hour of incubation at any concentration. As the concentration increased, the viability percentages dropped steadily. At concentrations of 6.25 mg/mL or higher for both KBLMt and KBLM, viability was completely lost. The negative control, on the other hand, had a revival rate of 45% ± 1.45% and a viability rate of 67%. The positive control (nonoxynol-9, 1 mg/mL) had a revival and viability rate of 0%.

**TABLE 2:** Sperm revival and viability of total methanolic extract and methanolic fraction of *Flueggea virosa*.

Extract	Concentration (mg/mL)	Sperm revival after 1 hour (%)	Sperm viability (%)
KBLMt	1.62	0 ± 0.00	25.5
	3.12	0 ± 0.00	15.00
	6.25	0 ± 0.00	0.00
	12.5	0 ± 0.00	0.00
	25.0	0 ± 0.00	0.00
	50.0	0 ± 0.00	0.00
KBLM	1.62	0 ± 0.00	23.0
	3.12	0 ± 0.00	16.5
	6.25	0 ± 0.00	0.00
	12.5	0 ± 0.00	0.00
	25.0	0 ± 0.00	0.00
	50.0	0 ± 0.00	0.00
Negative control	​	45 ± 4.5	67.00 ± 8.6
N-9 (1 mg/mL)	​	0 ± 0.00	0.00

KBLMt; Total methanolic extract, KBLM; methanolic fraction, N-9; Nonoxynol-9, positive control.

### 3.3 *In vitro* functional assessment of sperm penetration and acrosin-related activity of *Flueggea virosa* extract (KBLMt) and fraction (KBLM)

The total methanolic extract (KBLMt) and the most active fraction (KBLM) were then subjected to the cervical mucus penetration and acrosin inhibition activity assays to further characterize their effects on sperm function.

#### 3.3.1 Cervical mucus penetration

KBLMt and KBLM reduced sperm penetration distance and velocity through simulated cervical mucus in a concentration-dependent manner (n = 3) (Figure 3). For KBLMt, penetration distance decreased from 6.00 ± 1.00 mm (6.3 mg/mL) to 2.70 ± 0.30 mm (50 mg/mL), corresponding to inhibition ranging from 66.67% to 88.89%. KBLM exhibited stronger activity, with penetration distance reduced to 1.33 ± 0.33 mm at 12.5 mg/mL (94.44% inhibition) and 7.00 ± 0.58 mm at 1.62 mg/mL (55.56% inhibition). The negative control (normal saline) showed the highest penetration distance (17.67 ± 0.88 mm) and velocity (5.000 μm/s), indicating unrestricted sperm migration. The positive control, nonoxynol-9 (1.0 mg/mL), reduced penetration to 10.33 ± 1.667 mm (44.44% inhibition). Both KBLMt and KBLM produced greater inhibition of sperm penetration than the positive control at comparable test conditions.

**FIGURE 3:**
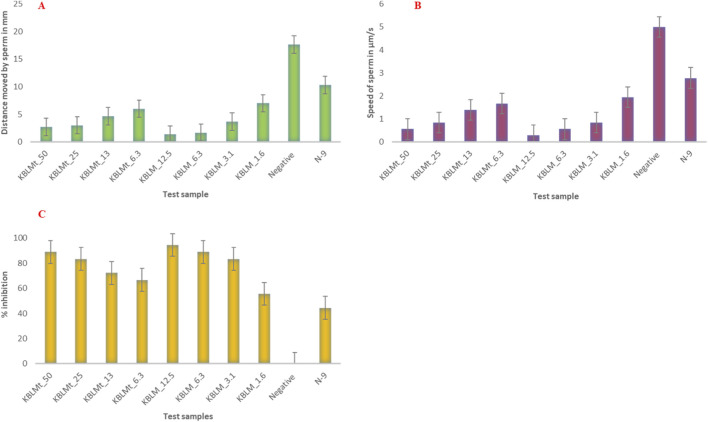
Effect of Flueggea virosa root bark extracts and fractions on sperm migration through cervical mucus. **(A)** Distance traveled by spermatozoa through cervical mucus following treatment with KBLMt and KBLM at the indicated concentrations (for example, KBLMt_50 and KBLM_25), where the letters denote the fraction and the number indicates the concentration (mg/mL). **(B)** Sperm velocity during penetration through cervical mucus under the same treatment conditions. **(C)** Percentage inhibition of sperm penetration through cervical mucus relative to the negative control (0.9% NaCl). Nonoxynol-9 (N-9) was included as a positive control.

#### 3.3.2 Acrosin inhibition activity

Acrosin activity (µIU/10^6^ sperm; mean, n = 3) was assessed after 3 h incubation in a semen-based assay and interpreted using the Kennedy et al. (1989) criteria (<14 inhibited; 14-25 equivocal; >25 not inhibited) (Table 3). KBLMt showed inhibited acrosin activity at 50 mg/mL (9.544) and 25 mg/mL (13.23), an equivocal response at 13 mg/mL (22.15), and no inhibition at 6.3 mg/mL (40.92). KBLM produced inhibited acrosin activity at all tested concentrations, with values of −2.86 (12.5 mg/mL), −1.773 (6.3 mg/mL), 11.58 (3.1 mg/mL), and 13.27 µIU/10^6^ sperm (1.62 mg/mL). The negative control (normal saline) exhibited an acrosin activity of 47.36 µIU/10^6^ sperm, consistent with no inhibition, while the positive control (benzamidine-HCl, 500 mM) showed complete inhibition (0 µIU/10^6^ sperm).

**TABLE 3:** Effect of F. virosa root bark extract and fraction on human sperm acrosin-related activity.

Test sample	Concentration (mg/mL)	µIU/10^6^ sperm	Acrosin activity
KBLMt	50	9.544	Inhibited
	25	13.23	Inhibited
	13	22.15	Equivocal
	6.3	40.92	Not inhibited
KBLM	12.5	−2.86	Inhibited
	6.3	−1.773	Inhibited
	3.1	11.58	Inhibited
	1.62	13.27	Inhibited
Negative control	-	47.36	Not inhibited
Positive control	1	0	Inhibited

Values are expressed as mean (n = 3). KBLMt: total methanolic extract; KBLM: methanolic fraction; N-9: nonoxynol-9 (positive control); negative control: sperm in normal saline. Acrosin inhibition is presented as acrosin activity (µIU/10^6^ sperm) after 3 h incubation.

### 3.4 Intravaginal *in vivo* efficacy and safety evaluation using a rabbit model

#### 3.4.1 Intravaginal *in vivo* safety evaluation

Histological assessment of rabbit cervicovaginal tissue showed a score of 0 for all evaluated endpoints in the saline control group (Table 4). In the KBLM-treated groups, the 3.9 mg/mL dose recorded a score of 0 for epithelial damage, leukocyte infiltration, edema, congestion/hemorrhage, and necrosis. At 7.8 mg/mL, mild epithelial damage and mild edema were observed (score 1 each), while leukocyte infiltration, congestion/hemorrhage, and necrosis remained absent (score 0). At 15.6 mg/mL, mild epithelial damage, mild leukocyte infiltration, and mild edema were recorded (score 1 each), with no congestion/hemorrhage or necrosis (score 0). In the N-9-treated group, all evaluated histological parameters were marked, with a score of three for epithelial damage, leukocyte infiltration, edema, congestion/hemorrhage, and necrosis. Representative histopathological sections were consistent with the histology scores, showing intact epithelium at 3.9 mg/mL, mild tissue alterations at 7.8 and 15.6 mg/mL, no observable lesions in the saline control, and extensive epithelial erosion and necrosis in the N-9 group (Figure 4).

**TABLE 4:** Representative summary of histology scores by treatment group (0-3 scale) for cervicovaginal tissue.

Endpoint/Concentration	Controls		KBLM (mg/mL)		
	Saline control	N-9 (20 mg/mL)	3.9 (low dose)	7.8 (middle dose)	15.6 (high dose)
Epithelial damage	0	3	0	1	1
Leukocyte infiltration	0	3	0	0	1
Edema	0	3	0	1	1
Congestion/Hemorrhage	0	3	0	0	0
Necrosis	0	3	0	0	0

Scores: 0 = none; 1 = mild; 2 = moderate; 3 = marked.

**FIGURE 4:**
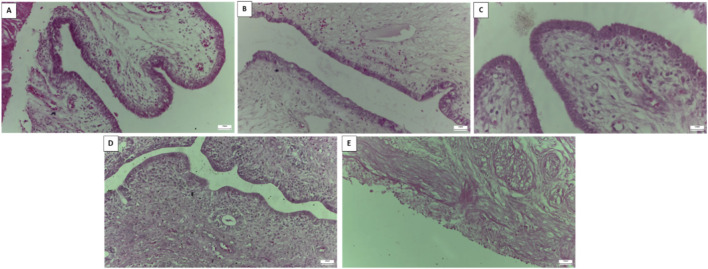
Representative histopathological sections of rabbit cervicovaginal tissue following intravaginal administration of KBLM and controls. **(A)** KBLM, 15.6 mg/mL (high dose), showing mild epithelial disruption, mild edema, and mild leukocyte infiltration, without congestion/hemorrhage or necrosis. **(B)** KBLM, 7.8 mg/mL (middle dose), showing mild epithelial degeneration and mild edema, with no leukocyte infiltration, hemorrhage, or necrosis. **(C)** KBLM, 3.9 mg/mL (low dose), showing intact epithelium with no edema, leukocyte infiltration, hemorrhage, or necrosis. **(D)** Normal saline-treated tissue showing intact epithelium with no histopathological lesions. **(E)** Nonoxynol-9, 20 mg/mL, showing complete epithelial erosion, pronounced edema, leukocyte infiltration, diffuse hemorrhage with clots, and confluent necrosis.

#### 3.4.2 Intravaginal *in vivo* efficacy evaluation

KBLM prevented pregnancy in a small rabbit proof-of-concept study at all tested doses (15.6, 7.8, and 3.9 mg/mL), with no pregnancies recorded in any treated group (0/5, 0%) and no fetuses observed per female (mean ± SD, 0.0 ± 0.0; median [range], 0 [0–0]). A similar outcome was observed in the positive-control group treated with nonoxynol-9 (20 mg/mL), in which no pregnancies occurred (0/5, 0%) and no fetuses were detected. In contrast, all animals in the negative-control group treated with normal saline became pregnant (5/5, 100%), with a mean of 5.2 ± 0.8 fetuses per female and a median of 5 [4-6] (Table 5).

**TABLE 5:** Intravaginal contraceptive efficacy of KBLM in rabbit model (n = 5/group).

Treatment	Dose (mg/mL)	n (females)	Pregnant/Total (%)	Fetus per female (mean ± SD)	Fetus per female (median [range])
KBLM	15.6	5	0/5 (0%)	0.0 ± 0.0	0 [0–0]
KBLM	7.8	5	0/5 (0%)	0.0 ± 0.0	0 [0–0]
KBLM	3.9	5	0/5 (0%)	0.0 ± 0.0	0 [0–0]
Positive control (Nonoxynol-9)	20	5	0/5 (0%)	0.0 ± 0.0	0 [0–0]
Negative control (normal saline)	-	5	5/5 (100%)	5.2 ± 0.8	5 [4-6]

Pregnancy was assessed by gross examination at necropsy. Litter size is reported as number of fetuses per female. n = 5 females per group.

### 3.5 Liquid chromatography-mass spectrometry (LC-MS/MS)

To describe the major putative chemical features, the KBLM fraction was profiled by using LC-MS/MS. The retention time (RT), molecular ions (m/z) and fragmentation patterns enabled the annotation of nine prominent peaks (Table 6). However, these annotations are putative and only represent the fraction’s chemical fingerprint while not implying that any listed compound is responsible for the observed fraction-level activity.

**TABLE 6:** Putative LC-MS/MS annotations of prominent chemical features in the methanolic fraction of *Flueggea virosa* root bark (KBLM).

Peak No.	Mode	RT (min)	Molecular ion (m/z)	Compound	References
1	+	0.6137	291 [M + H]^+^	Catechin	Souleymane et al. (2023), Spáčil et al. (2010)
2	+	2.4689	303 [M + H]^+^	Quercetin	Jiang and Gates (2024), Souleymane et al. (2023)
3	+	6.3396	614 [M + H]^+^	Fluevirine E (dimeric indole alkaloid)	Gan et al. (2006), Yang et al. (2020)
4	+	7.8970	607 [M + H]^+^	Rutin (Quercetin-3-O-rutinoside)	Cuyckens and Claeys (2004), Mecenas et al. (2018)
5	+	8.0166	607 [M + H]^+^	Quercetin diglycoside isomer (neohesperidoside)	Lin et al. (2021), Souleymane et al. (2023)
6	+	8.2117	621 [M + H]^+^	Kaempferol 3-O-(4-galloyl)-β-D-glucopyranoside	Chen et al. (2022), Ojha et al. (2024)
1	-	2.113	443 [M–H]^-^	Proanthocyanidin trimer (catechin/epicatechin type)	Dias et al. (2012), Souleymane et al. (2023), Tala et al. (2013)
2	-	2.4803	599 [M–H]^-^	Unknown flavonoid glycoside	Akbaribazm et al. (2020), Sanogo et al. (2009)
3	-	2.8450	633 [M–H]^-^	Corilagin	Souleymane et al. (2023), Świątek et al. (2021), Wu et al. (2009)

+; Positive Mode, -: negative mode.

## 4 Discussion

This study provides preliminary proof-of-concept evidence that the methanolic fraction (KBLM) of *F. virosa* root bark has sperm-disrupting activity and showed intravaginal contraceptive activity in a rabbit model. In *in vitro* assays, KBLM rapidly immobilized sperm, reduced viability, impaired cervical mucus penetration and diminished acrosin-related activity. In a small rabbit proof-of-concept study, intravaginal administration of KBLM prevented pregnancy while demonstrating absent-to-mild short-term cervicovaginal changes, in contrast to the marked tissue injury observed with nonoxynol-9. While these preliminary results are positive, we limit our interpretation to the fraction as tested without ascribing activity to any LC-MS/MS-annotated metabolite.

The KBLM caused rapid, irreversible immobilization and viability loss of human sperm *in vitro*. At 1.62 mg/mL it eliminated all progressive motility, and even high-dose washing failed to restore movement. This behavior mirrors saponin-rich plant spermicides, which are known to permeabilize sperm membranes and cause cell death. For instance, saponins from *Saponaria officinalis* irreversibly immobilize sperm and abolish viability within minutes (Xu et al., 2022), and these compounds act by disrupting the sperm plasma membrane through exaggerated lipid permeabilization (Xu et al., 2022). By analogy, KBLM’s rapid “no-revival” effect and steep viability drop suggest a membrane or metabolism-disrupting mode of action, not merely transient capacitation blockade. In sum, KBLM behaves like a fraction with rapid sperm-immobilizing activity: it permanently paralyzes or kills sperm (as many herbal spermicides do) rather than just briefly stunning them (Sefrioui et al., 2021).

KBLM also impaired sperm function downstream of motility, as reflected by performance in simulated cervical mucus (SCM). At 1.62 mg/mL, treated sperm penetrated only 7 mm into the mucus (56% inhibition), and at 12.5 mg/mL penetration fell to 1.3 mm (94% inhibition). By comparison, N-9 at 1 mg/mL produced only 44% inhibition under the same conditions. This marked reduction in progressive movement implies that KBLM not only stops sperm motility but may also alter sperm-mucus interactions. *In vivo*, cervical mucus normally filters out slow or abnormal sperm, allowing only the most motile cells to progress (Lee et al., 2021). Thus, any agent that drastically reduces the pool of progressive sperm, as KBLM does, will directly decrease the number of sperm reaching the fertilization site. Indeed, Sefrioui et al. (2021) reported that N-9 significantly reduces both the percentage of progressively motile sperm and their penetration distance through mucus (Sefrioui et al., 2021). KBLM’s stronger inhibition of sperm penetration, relative to N-9, therefore suggests that it could be potentially effective at limiting sperm passage through the mucus barrier, a key contraceptive mechanism in natural conception.

KBLM markedly reduced acrosin-related activity in the semen-based BAPNA assay at concentrations ≥1.62 mg/mL. However, because this assay was performed in whole semen rather than with purified enzyme, the present data should be interpreted as functional evidence of reduced acrosin-related activity rather than definitive proof of direct, specific acrosin inhibition. Acrosin is a trypsin-like serine protease in the sperm acrosome that facilitates penetration of the oocyte’s zona pellucida. Blocking acrosin is known to prevent fertilization: *in vitro* studies show that acrosin-deficient sperm cannot penetrate the zona and therefore fail to fertilize (Quan et al., 2025). In our study, KBLM suppressed acrosin activity at doses far below those required for the crude extract, whereas KBLMt had minimal effect except at much higher concentrations (≥25 mg/mL). This means that KBLM-treated sperm are doubly handicapped: they are immotile and also enzymatically incapable of zona penetration. Critically, acrosin inhibition can be effective even if some sperm retain motility because it blocks the final step of fertilization (Quan et al., 2025). Thus, KBLM’s combination of motility arrest and acrosin inhibition may reduce the fertilizing capacity of surviving sperm.

The absence of pregnancy in the KBLM-treated groups provides initial *in vivo* support for the fraction-level observations. The complete pregnancy rate in the saline control group (5/5) confirms that the mating protocol was successful and that the female rabbits were reproductively competent under the study conditions. Nevertheless, the animal efficacy study was small and descriptive, and therefore cannot be used to conclusively infer definitive contraceptive efficacy, formal dose-response relationship, clinical relevance, or superiority over existing products. Larger controlled studies that include appropriate estrous-cycle control and use consistent formulations and repeated independent extract batches are required to confirm reproducibility.

Although following 10 days of daily intravaginal KBLM dosing at up to 15.6 mg/mL produced absent-to-mild cervicovaginal changes while nonoxynol-9 caused marked epithelial disruption, hemorrhage, inflammation and necrosis in this rabbit model, these short-term tolerability results should be conservatively interpreted. They indicate but not establish probable human vaginal safety of the fraction and as such, additional comprehensive safety evaluations are required. Dedicated studies using human vaginal and cervical epithelial models, tissue explants, microbiome compatibility assays, longer exposure periods, recovery and reversibility assessments, and local retention or pharmacokinetic studies are required (Lagunas-Cruz et al., 2026).

Several descriptive chemical fingerprints were determined in KBLM using LC-MS/MS profiling. Although putative phenolic, flavonoid, and tannin-related chemical features were revealed in the fraction, the observed activity cannot be ascribed to any of these components prior to further chemical characterization and determination of the mode of action. This is largely because phenolic and flavonoid scaffolds are notorious pan-assay interference compounds (Bolz et al., 2021; Magalhães et al., 2021). Accordingly, the present study does not attribute sperm immobilization, reduced acrosin-related activity, mucus-penetration inhibition, or rabbit contraceptive efficacy to any of the individual LC-MS/MS-annotated compounds. Future work using authentic standards, fraction re-testing, isolated constituents, counter-screening, and direct sperm-function assays should be undertaken to tease out compound-level activity.

Notably, the study findings distinguish KBLM from nonoxynol-9 in a rabbit screening model. Even though both prevented pregnancy under the experimental conditions, KBLM produced absent-to-mild short-term cervicovaginal changes whereas nonoxynol-9 caused marked epithelial disruption, hemorrhage, inflammation and necrosis. While this difference supports continued evaluation of KBLM as a fraction-level topical contraceptive candidate, it should not be interpreted as proof of clinical safety or superiority. Longer exposure studies, appropriate formulation, microbiome compatibility testing and reproductive-cycle assessments are needed to establish product translation potential of the fraction (Chopra et al., 2024; North et al., 2022).

The development of plant fractions as preclinical contraceptive candidates poses several challenges including batch variability, chemical complexity and need for suitable formulation. Here, KBLM was evaluated as a probable intravaginal contraceptive in a preliminary proof-of-concept biological screening. Consequently, formulation-critical properties such as pH, osmolality, viscosity, mucoadhesion, and vaginal retention were not characterized in the present work (Franz-Montan et al., 2026; Tasdighi et al., 2012). Future KBLM formulations should be buffered to an appropriate vaginal pH range and designed to avoid hyperosmolar or otherwise irritating vehicles. Overall, the fraction-level combination of sperm incapacitation, inhibition of mucus penetration, reduced acrosin-related activity, and lower short-term irritation compared to nonoxynol-9, supports further controlled preclinical evaluation of the fraction.

## 5 Conclusion

Collectively, our results obtained from functional assays and a pilot proof-of-concept study in a rabbit model have demonstrated sperm-disrupting activity and intravaginal contraceptive activity of the KBLM fraction. In addition to the disruption of multiple sperm functions *in vitro,* the blocking of pregnancy in a rabbit model occurred with absent-to-mild short-term local tissue changes in the rabbit model. Although several compounds were putatively annotated using LC-MS/MS, the majority of these are routinely found in natural products. Thus, an in-depth determination and characterization of the repertoire of chemical constituents and direct validation of their contribution to the observed fraction-level effects is required. Such studies are needed to determine whether any isolated constituent contributes specifically to the observed fraction-level activity. Until such validation is performed, no therapeutic relevance is assigned to any LC-MS/MS-annotated metabolite.

## 6 Study limitations and recommendations

Although the present study provides encouraging proof-of-concept evidence for the contraceptive potential of the methanolic fraction (KBLM) of *F. virosa* root bark, several limitations should be investigated in future in order to advance KBLM as an intravaginal contraceptive. First, the safety assessment was restricted to short-term intravaginal exposure over 10 days and therefore cannot be used to infer longer-term local tolerability, reversibility, tissue recovery, or chronic mucosal responses upon repeated use. We also acknowledge that larger, controlled studies, with appropriate estrous-cycle control, will be required to confirm efficacy and ascertain reproducibility of the effects. In addition, the cytotoxicity evaluation was limited to Vero cells, which only provide a preliminary indication of general mammalian cell safety and do not adequately represent the vaginal environment. Future studies should therefore incorporate human vaginal and cervical epithelial cell lines, *ex vivo* vaginal tissue models, and assessments of compatibility with beneficial vaginal microbiota. Moreover, the biological activity was demonstrated using a complex fraction rather than isolated pure compounds. The putative LC-MS/MS annotations include common phenolic/flavonoid chemical classes that may behave as PAINS; therefore, these features should not be treated as activity-bearing constituents without authentic-standard confirmation, counter-screening, and direct sperm-function assays. Further phytochemical analyses including isolation, purity assessment, authentic-standard confirmation, PAINS/counter-screening where appropriate and direct sperm-function assays of purified constituents are required for standardization and untangling of their mode of action. In particular, confirmation of the acrosin findings using purified enzyme preparations, inhibitor-specific controls and kinetic analyses to determine whether the observed effect reflects direct enzyme inhibition or broader disruption of acrosomal function ought to be performed.

## Data Availability

The original contributions presented in the study are included in the article/supplementary material, further inquiries can be directed to the corresponding authors.
